# Retrieval of Missing Spliced Leader in Dinoflagellates

**DOI:** 10.1371/journal.pone.0004129

**Published:** 2009-01-05

**Authors:** Huan Zhang, Senjie Lin

**Affiliations:** Department of Marine Sciences, University of Connecticut, Groton, Connecticut, United States of America; Baylor College of Medicine, United States of America

## Abstract

Spliced leader (SL) *trans*-splicing has recently been shown to be a common mRNA processing mechanism in dinoflagellates, in which a short (22-nt) sequence, DCCGUAGCCAUUUUGGCUCAAG (D = U, A, or G), is transplanted from the 5′-end of a small non-coding RNA (SL RNA) to the 5′ end of mRNA molecules. The widespread existence of the mechanism in dinoflagellates has been demonstrated by detection of this SL (DinoSL) in a wide phylogenetic range of dinoflagellates. Furthermore, the presence of DinoSL in the transcripts of highly diverse groups of nuclear-encoded genes has led us to postulate that SL *trans*-splicing is universal in dinoflagellate nuclear genome. However, some observations inconsistent to this postulation have been reported, exemplified by a recent article reporting apparent absence of DinoSL in the transcripts of some nuclear-encoded genes in *Amphidinium carterae*. Absence of SL in these gene transcripts would have important implication on gene regulation in dinoflagellates and utility of DinoSL as a universal dinoflagellate-specific primer to study dinoflagellate transcriptomics. In this study, we re-examined transcripts of these genes and found that all of them actually contained DinoSL. Therefore, results to date are consistent to our initial postulation that DinoSL occurs in all dinoflagellate nuclear-encoded mRNAs.

## Introduction

To the list of unusual molecular and cytological characteristics of dinoflagellate recognized in the last two decades was recently added another: these organisms possess a unique spliced leader (SL) *trans*-splicing machinery [1], [2]. SL *trans*-splicing is an mRNA processing mechanism, in which a short RNA fragment (i.e. SL, ∼15–50 nt) from a small non-coding RNA (SL RNA) is transplanted to a splice acceptor site in the 5′-untranslated region of independently transcribed pre-mRNAs. Through this process, mature mRNAs are formed with the SL sequence occupying the 5′ ends. SL RNA *trans*-splicing generally has a variety of functions: 1) generating translatable monocistronic mRNAs from polycistronic precursor transcripts; 2) sanitizing the 5′ end of mRNAs; 3) stabilizing mRNAs, and 4) possibly regulating gene translation (for reviews see: [3]–[5]). In dinoflagellates, the exact function of the SL-based *trans*-splicing remains to be studied although its involvement in functions 1, 2, and 4 mentioned above is very possible [1].

Dinoflagellates share SL *trans*-splicing with organisms such as Euglenozoa, nematodes, platyhelminthes, cnidarians, rotifers, ascidians, and appendicularia (for reviews see: [3], [5], [6]). However, the elements that make up the machinery in dinoflagellate seem to be distinct. The SL RNA generally contains two functional domains: an exon (i.e. SL) that is transferred to an mRNA and an intron that contains a binding site for ribonucleoprotein particle assembly (Sm) to facilitate splicing. In dinoflagellates, the SL sequence (DinoSL), DCCGUAGCCAUUUUGGCUCAAG (D = U, A, or G), though conserved in all dinoflagellate lineages examined to date, shows no similarity to counterpart in other organisms. Furthermore, SL gene transcript (i.e. SL donor RNA) in dinoflagellates is unusually short (50–60 bp). In addition, while the conserved Sm-binding site [Sm motif] in other organisms (RAU_4-6_GR in the kinetoplastids, freshwater planarians and *Caenorhabditis*, RAUUUUCGG in *Hydra*, AGCUUUGG in *Ciona*, AGCUUUUCUUUGG in *Schistosoma*, and AAYUYUGA in Rotifera ([1] and refs there in) usually is located in the intron of the SL RNA, dinoflagellate SL intron does not carry this Sm-binding site; instead a sequence (AUUUUGG) highly similar to the binding site exists in the exon. This observation suggests that either dinoflagellates use a unique Sm-binding site located in the intron, or the apparent Sm-binding site in the exon indeed functions in *trans*-splicing. These unusual features have prompted questions about whether truly functional SL RNA in dinoflagellates exists in other gene structures (longer SL RNA containing a Sm-binding site in the intron and genomic organization with 5S rDNA) but somehow escaped our detection, as shown for *Karenia brevis* [2]. Our reanalysis of the SL RNA gene and transcript structure for *K. brevis* and five other dinoflagellates provided an answer. Our new data indicated that the SL-5S genomic structure [2] indeed occurred as a second genomic structure in almost all dinoflagellate species we examined; however, only the SL RNA structure (short, lacking Sm-binding site in the intron) we reported initially [1] can be detected either on Northern blot or through rapid amplification of cDNA 3′ end of dinoflagellate SL RNA (Zhang et al. submitted). Thus, the proposition that SL RNA in *K. brevis* and probably other dinoflagellates contains a longer intron that possesses a Sm-binding site is not supported.

Recently, in a survey of genomic arrangements of genes in two dinoflagellate species, Bachvaroff and Place [7] analyzed genomic sequences and the corresponding cDNAs for many genes from dinoflagellate *Amphidinium carterae*. The authors have addressed several aspects about the genomic organization of genes in dinoflagellates and provided valuable evidence on gene arrangement, expression and spliceosomal introns for this important lineage of eukaryotes. They also examined the presence of DinoSL for cDNAs of 47 genes by PCR using DinoSL as a primer and found that approximately two thirds of these genes were *trans*-spliced. The authors further noted that almost all of the highly expressed genes were organized in tandem repeats and their transcripts were SL *trans*-spliced, whereas genes that they failed to detect SL on corresponding transcripts were expressed at lower levels. It was suggested that the transcripts of the highly expressed genes were more likely to be *trans*-spliced than less highly expressed genes, although the authors acknowledged that negative PCR results should be considered not significant [7]. This conclusion implies that DinoSL potentially marks only transcripts of highly expressed genes rather than all nuclear-encoded genes in dinoflagellates. Furthermore, if confirmed to be true, the conclusion would have significant implication in regard to the potential of DinoSL as a global marker of dinoflagellate nuclear-encoded transcripts to facilitate exclusive synthesis of dinoflagellate cDNAs in the presence of RNA from other organisms. Therefore, we attempted to address the issue by using newly obtained as well as previously published data. We experimentally analyzed ten of the twelve *A. carterae* nuclear-encoded genes suggested to be “non- *trans*-spliced” [7] and successfully detected DinoSL at the 5′ end of their transcripts. Together with previous data showing presence of DinoSL in transcripts of form II Rubisco and single-stranded DNA-binding replication protein A [1], the two other genes also suspected to lack SL [7], we demonstrated the presence of DinoSL in the transcripts of all the twelve genes, thereby reinstating the postulation that DinoSL occurs widely in dinoflagellate nuclear-encoded transcripts.

## Materials and Methods

### Preparation of RNA samples and cDNA syntheses

**Figure 1:**
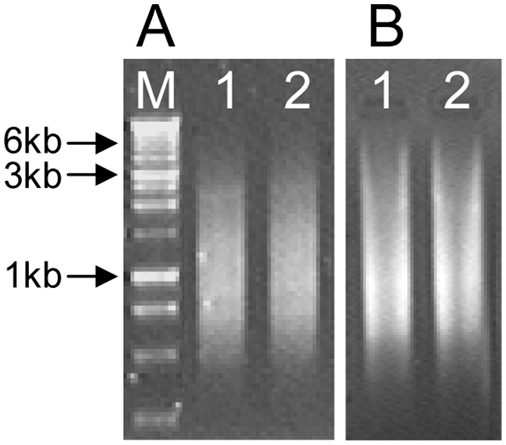
Agarose gel electrophoresis of SL-based full-length cDNA libraries of dinoflagellates. A) *Amphidinium carterae* (CCMP1314). B) *Karlodinium veneficum* (CCMP1975). First strand cDNA libraries were synthesized from 1 µg (lane 1) or 2.5 µg (lane 2) total RNA and used as templates for PCR amplification of full-length cDNAs with DinoSL-Racer3 as the primer set. Lane M, 1 kb DNA ladder.

Cultures of *A. carterae* (CCMP1314) and *Karlodinium veneficum* (CCMP1975, CCMP 2778) were grown in f/2 seawater medium at 20°C at a 12 h∶12 h light∶dark photocycle with a photon flux of approximately 75 µE·m^−2^ s^−1^. When the cultures were in the exponential growth phase, 10^6^ cells were harvested by centrifugation at 3000×g at 20°C and the cell pellet for each species was resuspended thoroughly in Trizol (Invitrogen) for RNA extraction [1]. Total RNA was extracted following our previous reports [1], [8], and the first-strand cDNA was synthesized with 1 µg and 2.5 µg total RNA, respectively, using GeneRacer Oligo dT primer (Invitrogen) and purified using DNA Clean-up & Concentrator (Zymo Research) [1]. cDNA equivalent to 50 ng and 250 ng total RNA were PCR-amplified using primer set DinoSL-Racer3 to enrich the full-length cDNAs (cDNAs with DinoSL and poly A tail). PCR was carried out using ExTaq (TaKaRa Mirus) under the following PCR program: 95°C 1 min for 1 cycle, followed by 95°C 20 sec, 72°C 2.5 min for 5 cycles, 95°C 20 sec, 65°C 30 sec, 72°C 2 min for 5 cycles, 95°C 20 sec, 60°C 30 sec, 72°C 2 min for 5 cycles, and 95°C 20 sec, 58°C 30 sec, 72°C 2 min for 15 cycles. PCR products were electrophoresized in a 1.2% agarose gel (Fig. 1) to confirm the cDNA quality, and then ligated into a T-vector. The ligates were transformed into competent cells, the resultant colonies were randomly picked up, and their plasmids were isolated and sequenced as previously reported [1].

### Primer design and PCR amplification of target genes and sequence analyses

In the previous study [7], 15 out of 46 *A. carterae* genes studied were suggested to be non- *trans*-spliced. These ‘non- *trans*-spliced’ genes included one mitochondrial gene (*coxIII*), two genes with identical name (violaxanthin deepoxidase), and one gene with unclear evolutionary source named as *Ectocarpus silicosus* virus (ESV). Among these genes, PCR amplification for the genomic complement of the ESV's EST was unsuccessful, raising question on its origin. In regard to *coxIII*, we have demonstrated in our previous study that dinoflagellate mitochondrial genes are not *trans*-spliced [1]. Therefore, in this study we excluded *coxIII*, one of the violaxanthin deepoxidase genes without a GenBank accession number, and ESV from further analysis. In addition, form II Rubisco and replication protein have already been shown to contain DinoSL from the other dinoflagellates *Prorocentrum minimum* (DQ884420) and *Pfiesteria piscicida* (DQ864840), respectively [1]. It is reasonable to expect that DinoSL also occurs in the transcripts of these two genes in *A. carterae*; therefore, no further analysis was done on these two genes here. For the remaining 10 potential ‘non- *trans*-spliced’ genes, specific reverse primers were designed based on gene sequences reported previously [7], [9] using Beacon Designer 3.0 (PREMIER Biosoft) (Table 1).

**Table 1:** Primers used in this study.

Primer name	Sequence (5′-3′)	Reference and application
DinoSL	DCCGUAGCCAUUUUGGCUCAAG (D = U, A, or G)	Forward primer for dinoflagellate full-length mRNA; [1]
Racer3	TGTCAACGATACGCTACGTAACG	Reverse primer for dinoflagellate full-length mRNA; [1]
Aca-AHCYR1	CCTGTGGCTGATGTGAAGATGT	Reverse primer for adenosylhomocysteinase; this study
Aca-AHCYR2	ACCAATCATCACATCCGTCGC	Reverse primer for adenosylhomocysteinase; this study
Aca-APX-R1	CAGCAACACGCAACACACAT	Reverse primer for ascorbate peroxidase; this study
Aca-APX-R2	TGAAGATAGATGCTGCGGATCG	Reverse primer for ascorbate peroxidase; this study
Aca-ACTR1	TCACAGTATTCAGTAATCGCTTCAC	Reverse primer for aspartate carbamoyltransferase; this study
Aca-ACTR2	AGTGATGGTCTCGTTCTTCTGAA	Reverse primer for aspartate carbamoyltransferase; this study
Aca-RBMR1	CACAGTTATCCGCCGTCCAT	Reverse primer for RNA binding motif; this study
Aca-RBMR2	TCCGATGAAGAGGTCACAACG	Reverse primer for RNA binding motif; this study
Aca-VDER1	AAGCACATACCAATCCTCGTCAA	Reverse primer for violaxanthin de-epoxidase; this study
Aca-VDER2	CTCTTGAGTCTTGGCAGGCG	Reverse primer for violaxanthin de-epoxidase; this study

First-strain cDNAs were used as PCR template. DinoSL was used as the forward primer paired with the gene specific reverse primers (Table 1). Two rounds of touch-down PCR were carried out with primer set DinoSL-R1 (first round PCR) and DinoSL-R2 (second round PCR) under the following conditions: 95°C 20 sec, 62°C 30 sec, 72°C 40 sec for 5 cycles; 95°C 20 sec, 58°C 30 sec, 72°C 40 sec for 30 cycle; 72°C 5 min for 1 cycle. For the 2^nd^ round of PCR, 100-fold diluted first round PCR amplicons were used as the template. PCR products were electrophoresized in 1.2% agarose gel, DNA bands were recovered using Zymoclean Gel DNA Recovery Kit (Zymo Research) and either directly sequenced or cloned into a TA vector [1]. In cases where PCR products were cloned, four resulting clones were randomly picked up and sequenced using BigDye Terminator v3.1 and analyzed on ABI 3730 DNA Sequencer (Applied Biosystems). The obtained sequences were aligned with the reported genomic as well as cDNA sequences (Bachvaroff and Place 2008; Bachvaroff et al. 2004) using CLUSTAL W (1.8) [10].

## Results

### Wide cDNA size range in the SL-based full-length cDNA libraries for *Amphidinium carterae* and *Karlodinium veneficum*

To address whether DinoSL only occurs in a selection of cDNAs, we ran a subsample of the full-length cDNA libraries on the agarose gel to examine whether the libraries were biased toward certain molecular weight range or discrete molecular size bands. Our result showed that the cDNA library was a continuous smear, with a molecular weight range 0.5 kb up to 6 kb (Fig. 1), as usually seen for a good quality cDNA library. Several hundred of the cDNA clones were sequenced, either from both ends or in fewer cases only from 5′-end, and showed highly diverse functional groups of genes [ref 1 and data not shown].

### Use of DinoSL led to successful PCR amplification of target genes

Using *A. carterae* cDNA library as the template, DinoSL as the forward primer and gene specific primers as the reverse primers, we successfully PCR-amplified the 5′-end region of the cDNAs for the five genes whose GenBank accession numbers were mentioned in [7] (Table 2). For the other five reported genes with no GenBank accession numbers given [7], we obtained cDNAs with the same gene names by randomly sequencing clones from *A. carterae* (1 cDNA) and *K. veneficum* (4 cDNAs), and BLASTing against GenBank database (Table 2).

**Table 2:** *Amphidinium carterae* gene transcripts previously reported to lack DinoSL and the corresponding cDNAs with DinoSL obtained in our laboratory.

Genes	GenBank accession nos of the genes (or cDNA) in previous report showing absence of DinoSL in cDNAs^a^	GenBank accession nos of cDNAs detected for *A. carterae* in the present study containing DinoSL	cDNAs with DinoSL detected in other dinoflagellates
Adenosylhomocysteinase	EU742862	FJ381675^c^	
Ascorbate peroxidase	EU742799	FJ381676^c^	
Aspartate carbamoyltransferase	CF066758	FJ381677^c^	
RNA binding motif	EU742819	FJ381678^c^	
Violaxanthin de-epoxidase	EU742815	FJ381679^c^	
U2 snRNP auxiliary factor	N/A	FJ381680^c^	
Rubisco	N/A		*Prorocentrum minimum* DQ884420^b^
Replication protein	EU742798		*Pfiesteria piscicida* DQ864840^b^
Axoneme protein	N/A		*Karlodinium veneficum* FJ381681^c^
ChlD	N/A		*Karlodinium veneficum* FJ381682^c^
Ketoacyl-reductase like	N/A		*Karlodinium veneficum* FJ381683^c^
pfsec61	N/A		*Karlodinium veneficum* FJ381684^c^

a Bachvaroff and Place 2008 [7].

b Zhang et al. 2007 [1].

c This study.

**Figure 2:**
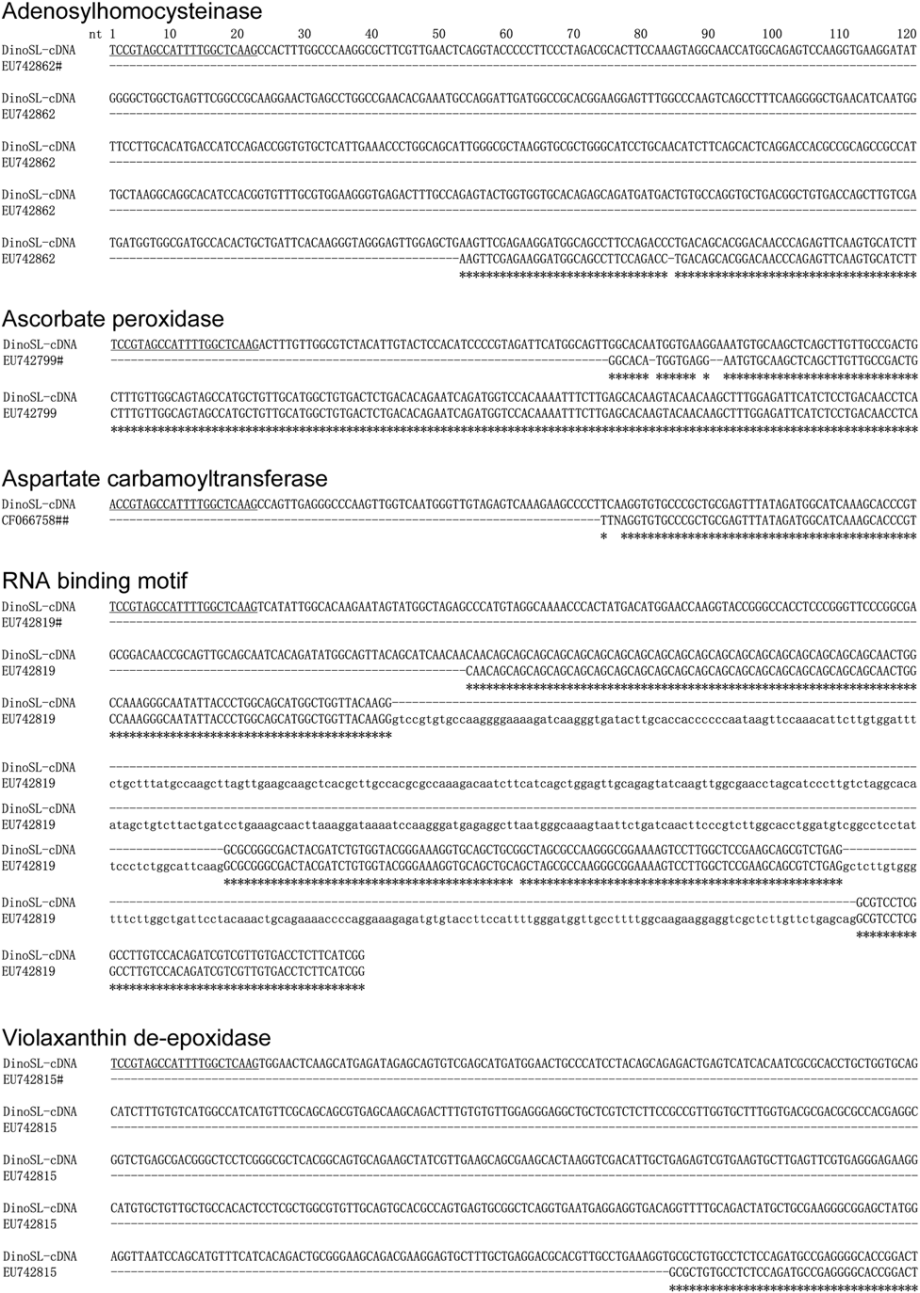
Alignments of the DinoSL-containing cDNAs obtained in this study (DinoSL) with their corresponding genomic (#) or cDNA sequences (##) reported previously [7], [9]. Exons are shown in upper case while introns in lower case; consensus positions are denoted by asterisks. The 22-nt DinoSL was underlined. Note that in all cases, the previously reported sequences missed varying lengths of sequences at the 5′-end.

Comparing the five DinoSL positive cDNAs we obtained (adenosylhomocysteinase, ascorbate peroxidase, aspartate carbamoyltransferase, RNA binding motif and violaxanthin de-epoxidase) with counterparts reported previously [7], [9], we found that these sequences missed 70–500 bp at the 5′-end region including DinoSL in those previous reports (Fig. 2).

## Discussion

It is at least equally difficult to prove that SL does not exist in some transcripts than that SL occurs in all transcripts in dinoflagellates. Until a complete transcriptome is sequenced, support for the latter can come from indirect evidence such as unbiased cDNA profiles and existence of SL in randomly examined genes. In contrast, proof of absence demands experiments carried out very carefully with proper controls to rule out false negative results, which is not easy to achieve. Because DinoSL can only be retrieved from an mRNA with intact 5′ end, examining whether a gene transcript is SL-*trans* spliced requires the isolation of intact mRNA and the construction of a good quality (enriched full-length) cDNA library. Many dinoflagellates contain strong inhibitors which will inhibit either reverse transcriptase or Taq DNA polymerase (Zhang and Lin, unpubl data). Probably for this reason, very few of the reported dinoflagellate ESTs contain DinoSL [1]. Only after testing many conditions had we successfully established a protocol for dinoflagellate RNA isolation/purification and cDNA construction [1], [8]. This protocol has allowed us to effectively isolate a large number of full-length cDNAs (with DinoSL at the 5′-ends) from various dinoflagellate species [ref 1 and Zhang and Lin, unpubl data]. As an example shown in Fig. 1, the full-length enriched cDNA libraries constructed based on this protocol showed smear of cDNAs with a wide range of molecular weights, suggesting no noticeable bias of DinoSL on types of cDNAs. Furthermore, our previous random sequencing of the libraries also demonstrated presence of DinoSL in highly diverse functional groups of gene transcripts [1]. Lidie and van Dolah [2] further reported SL RNA *trans*-splicing for a number of different genes in *K. brevis*. The detection in the present study of DinoSL at the 5′ end of the gene transcripts recently reported to escape SL RNA *trans*-splicing [7] has provided additional supporting evidence for the ubiquity of DinoSL in dinoflagellate nuclear gene transcripts. The failure of detecting DinoSL for cDNAs of the twelve genes in the previous study [7] could have stemmed from truncated mRNAs isolated leading to synthesis of cDNAs missing DinoSL and even adjacent 5′ untranslated region, or from existence of inhibitors in the cDNA libraries causing failure of PCR amplification.

The retrieval of the missing DinoSL from the cDNAs of these genes indicates that so far there is no direct evidence of absence of DinoSL in dinoflagellate nuclear-encoded gene transcripts. While no DinoSL has been detected for chloroplast- and mitochondrial-encoded gene transcripts [1], the results of previous and the present studies suggest that likely SL is present in all nuclear-encoded transcripts in dinoflagellates. Because DinoSL sequence is different from SL sequences in other organisms so far shown to harbour the spliced leader *trans*-splicing and BLAST analysis essentially showed no match to other gene sequences [1], the results to date suggest that DinoSL is unique in dinoflagellates relative to other *trans*-splicing organisms and universal within dinoflagellate phylum. Hence, DinoSL can be used to separate and amplify dinoflagellate nuclear-encoded full-length cDNAs from a mixed RNA sample extracted from different organisms using the methods developed [ref 1, Zhang and Lin, unpubl data]. For instance, when studying a heterotrophic dinoflagellate such as *Pfiesteria piscicida* fed with another microalga, presence of the prey alga has always posed a problem to attempts to isolate cDNAs of the grazer dinoflagellate without interference of prey cDNAs. Even when the prey concentration decreases to a low level as a result of grazing, cDNAs originated from the prey alga *Rhodomonas* sp., such as photosynthesis related genes, could still be detected from the cDNA libraries constructed from the *P. piscicida* culture (Zhang and Lin, unpubl data). With DinoSL, cDNA from the grazer dinoflagellate can be isolated regardless the prey alga is present or not and at what abundance [1]. More importantly, to gain insights into what dinoflagellates are doing in the natural environment, isolating dinoflagellate cDNAs from a natural plankton sample, where numerous other planktonic microbes coexist, is of paramount importance but it has been impossible. Now with the use of DinoSL as a selective primer, dinoflagellate *in situ* gene expression can be studied without the interference of coexisting plankton. Further verification of the ubiquity of DinoSL in dinoflagellates and validation of using DinoSL to profile dinoflagellate *in situ* transcriptome (meta-transcriptome) are underway in our laboratory.
